# Digitalized long-lasting insecticidal nets mass distribution campaign in the context of Covid-19 pandemic in Kongo Central, Democratic Republic of Congo: challenges and lessons learned

**DOI:** 10.1186/s12936-022-04258-8

**Published:** 2022-09-01

**Authors:** Joris Losimba Likwela, Phillipe Lukanu Ngwala, Albert Kalonji Ntumba, Deogratias Cibinda Ntale, Eric Mukomena Sompwe, Godé Kanyeba Mpiana, Joseph Kalonji Tshula, Tathy Kalonda Likwela, Patrick Kanku-Ka-Munabe, Adrien N.’siala Kumbi, Gilbert Kulimushi Ndahambara, Helen Cibinda Ntale, Michele Luntadila Kiamenga, Jules Kabongo Njila, Ghislain Makhan Yav, Didier Gasigwa Baneti, Julian Austin

**Affiliations:** 1 University of Kisangani, Kisangani, Democratic Republic of the Congo; 2 Sanru Asbl, Kinshasa, Democratic Republic of the Congo; 3 TIPTOP Project, Jhpiego DR of the Congo, Kinshasa, Democratic Republic of the Congo; 4 National Malaria Control Programme, Kinshasa, Democratic Republic of the Congo; 5 School of Public Health, University of Lubumbashi, Lubumbashi, Democratic Republic of the Congo; 6 Health and Wellbeing, Torus Foundation, Liverpool, UK; 7 Cellule d’Appui à la Gestion Financière, Ministère de la Santé, Kinshasa, Democratic Republic of the Congo; 8 Against Malaria Foundation, London, UK

**Keywords:** LLIN Campaign, Covid-19, Digitalization, Lessons, Democratic Republic of Congo

## Abstract

**Background:**

The Democratic Republic of the Congo (DRC) organized a first mass distribution campaign of long-lasting insecticidal nets (LLINs) with digitalized data management with coordinated support from the Ministry of Health (MOH) and Santé Pour Tous En Milieu Rural—an ‘Association sans but lucratif’ (SANRU Asbl), in the context of the Covid-19 pandemic in Kongo Central province. This article describes the planning and implementation process of this campaign as well as the challenges and lessons learned.

**Methods:**

The planning and implementation process was performed in line with the standard guidance issued by the National Malaria Control Programme (NMCP) following the start of Covid-19. The changes and adaptations put in place as well as the challenges encountered are described.

**Results:**

A total of 5,629,211 people were registered (7.7% above projection) in 1,065,537 households (6.2% below projection) giving an average of 5.3 people per household. Of a total of 3,062,850 LLINs ordered, 2,886,096 were distributed to households (94%). Out of 11,070 villages and 3,947 teams planned, 91.7% of villages were reached and 93% of teams were established.

**Conclusion:**

The revision of standards of campaign implementation during Covid-19, as well as effective coordination supported by real-time decision-making through digital data management, have been factors in the success of this campaign. Maintaining this momentum is essential to ensure the continuity of malaria prevention services for the population.

## Background

Malaria remains a serious threat to development in intertropical countries, in spite of a reduction in malaria cases estimated to 229 million cases in 2019 from 238 million in 2000. The Democratic Republic of the Congo (DRC) is the second most affected country in the world, accounting for about 12% of global cases and is among the 29 countries that bear 95% of the global malaria burden. The occurrence of the Covid-19 pandemic has caused dysfunction within the health system that could lead to higher-than-expected malaria-related morbidity and mortality [1].

Vector control with long-lasting insecticidal nets (LLIN) is one of the key strategies for malaria prevention and has contributed to a decrease in malaria worldwide [2, 3]. The World Health Organization (WHO) recommends universal coverage of at-risk populations through mass distribution campaigns. The use of LLIN has increased sharply in sub-Saharan Africa over the past decade, resulting in a significant decrease in malaria morbidity and mortality [4–7]. Over the past 3 years however, progress has stagnated, possibly due, in part, to the emergence of vector resistance to insecticides [1, 8–10].

The outbreak of the Covid-19 pandemic has occupied the political and health agendas of all countries, raising fears of a devastating impact on other disease control programmes, including malaria [11]. The WHO stressed the importance of maintaining malaria prevention, diagnosis and treatment activities [12]. Published modelling projects a risk of increasing the burden of malaria by up to 36% compared to a context without COVID19 with a potential return to malaria mortality levels last observed in 2000 [13, 14].

In the DRC, the Covid-19 epidemic was declared on 10th March 2020 [15] and the response measures resulted in the suspension of all mass distribution campaigns planned for 2020, planned to cover about 59 million people in 14 over 26 provinces in the country. Considering that for every 1000 children sleeping under LLIN in areas of intense malaria transmission, as is the case in almost the entire DRC, there would be nearly 6 lives saved [7], there is therefore reason to estimate more than 70,000 preventable deaths of children under 5 years of age in 2021.

Based on the guidance of The Alliance for Malaria Prevention (AMP) [16], the National Malaria Control Programme (NMCP) and its partners organized a series of workshops to review the planning standards and implementation of LLIN mass distribution campaigns in the context of Covid-19 as well as advocacy sessions bringing together the Civil Society with the Covid-19 Multi-Sector Response Committee (CMR COVID19) with an aim of restarting campaigns in the second semester of 2020 and completing in Q1 2021. Kongo Central is one of 10 provinces selected for the WHO-recommended "High Burden, High Impact" (HBHI) approach [17] to accelerate the impact of malaria control [18] and was identified to pilot the digitalized campaign data management approach for the first time at the provincial level with coordinated support from the MOH and SANRU Asbl.

This article describes the process of planning and implementing the LLIN mass distribution campaign in Kongo Central in 2020 with digitalized data management. The aim is to share this experience of in the context of the covid-19 pandemic.

## Methods

### Summary description of the province

**Fig. 1:**
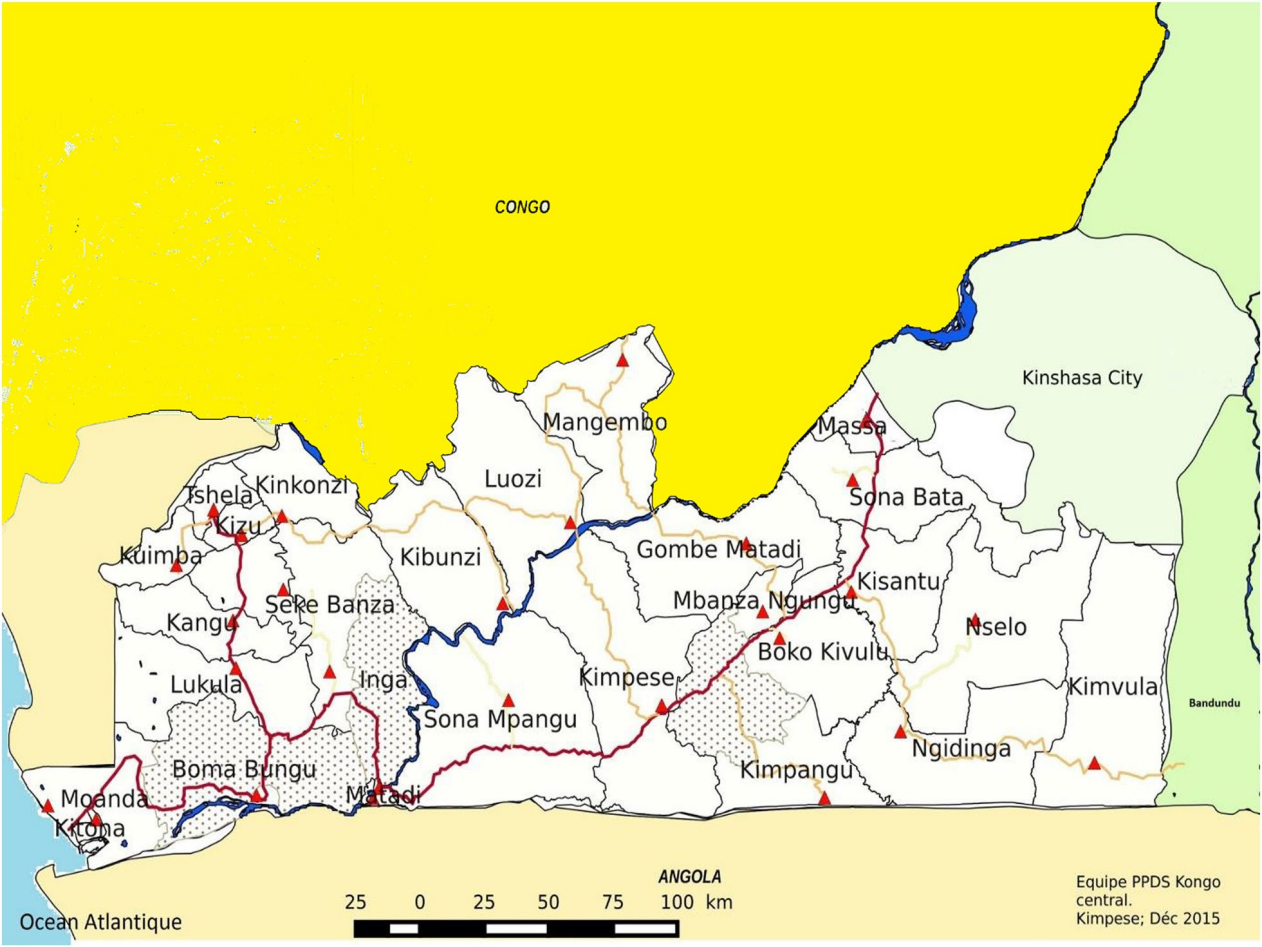
Health map of Central Kongo Province.  No General hospital in the health district,  General referral hospital, Main roadway, Secondary roadway,  tertiary roadway,  aquatic area

Kongo Central is one of 26 provinces in the DRC with an estimated population of 5,575,000 in 2015 [19]. For mass campaign activities, registration is carried out taking into account the dynamics of population growth. Thus, for this campaign, the population registered during the LLIN distribution campaign in 2017 estimated at 5,225,725 inhabitants was used, applying an annual rate of increase of 2.9% according to the natural increase estimates of the “Institut National de Statistique” (INS) [17]. The province is divided into 31 health districts (HD), including 3 urban and 28 rural (Fig. 1). These HDs are divided into 399 health areas (HA).

In this province, malaria is endemic with perennial transmission [20]. The entire population is at risk of malaria infection and malaria is the leading cause of morbidity and mortality. In 2019, the reported incidence of the disease was 327 cases per 100,000 populations with a hospital mortality of 40 deaths per 100,000 populations attributed to malaria [21].

#### The process of implementing the campaign

**Fig. 2:**
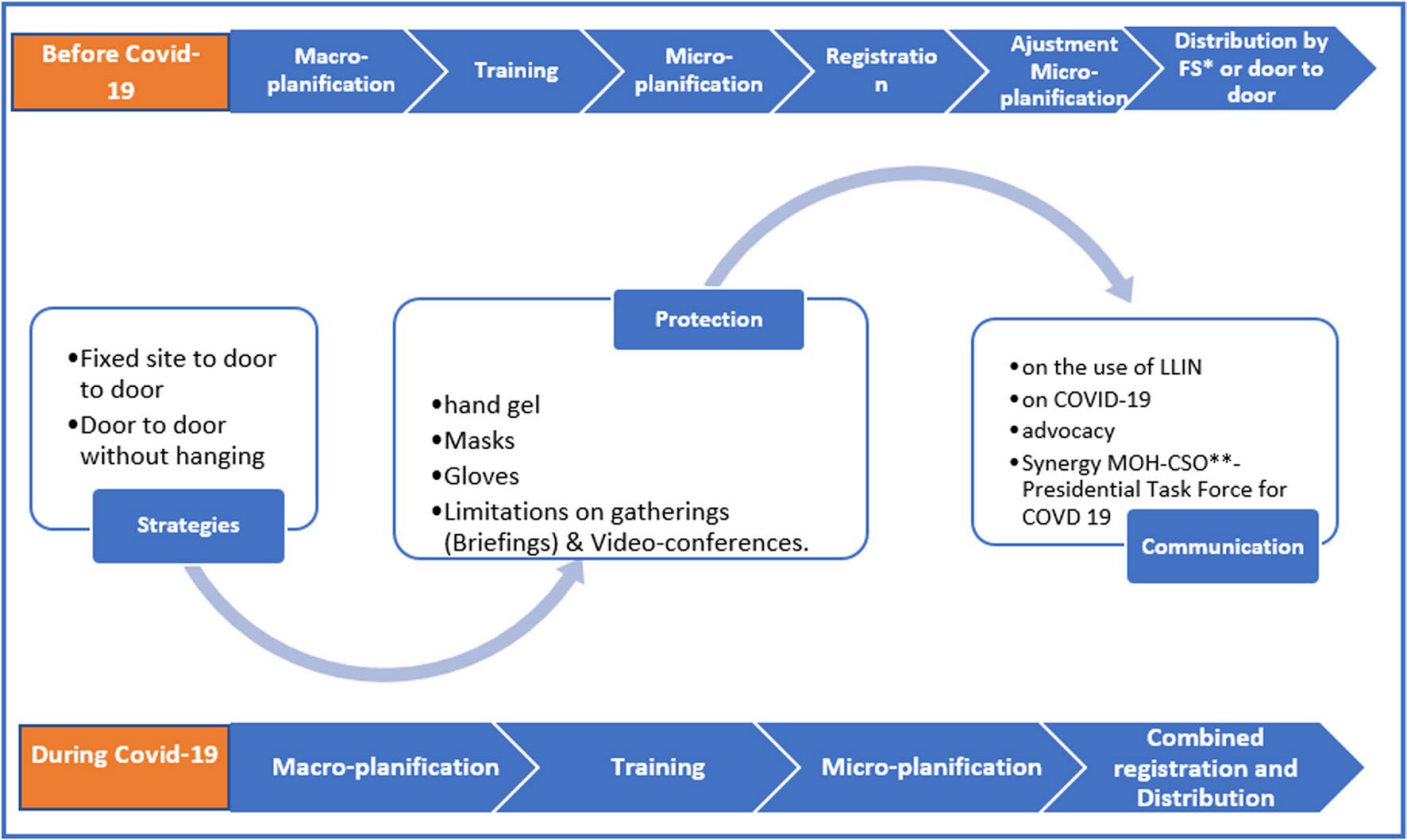
Changes to the campaign organizing process at the time of Covid-19. *Fixed Sites, **Civil Society Organization.

The process of the 2020 LLIN distribution campaign was determined by the occurrence of the Covid-19 pandemic, which caused a great deal of upheaval. The outbreak of the disease of Covid-19 was declared on 10th March 2020 in the DRC in Kinshasa, and since then the disease has spread to a total of 23 of the country’s 26 provinces. Central Kongo ranked 4th with 1625 cases out of a cumulative number of 27,468 cases and 726 deaths in the country [15]. In order to take this into account, the NMCP and its partners had to revise the organizational strategies of the LLIN distribution campaign (Fig. 2) [22].

As part of the Global Fund to Fight AIDS, Tuberculosis and Malaria (GFTAM) grants, the country had two distribution strategies: fixed site and door-to-door with LLINs being fixed in sleeping spaces by the distribution teams. In both cases, distribution was organized after registration to provide robust demographic data. In order to limit contact between teams and households, there has been a standardization of approaches by bringing all distributions back to the door-to-door method without putting up LLIN’s in houses.

Unlike previous distributions where registration preceded distribution, the registration was combined with distribution to reduce the number of contacts between Community Health Workers (CHW) and households and thus avoid the risk of increased transmission of Covid-19. The teams were equipped with hand gel and masks. The person in charge of handing over LLINs to households was wearing gloves to avoid possible irritation that may occur as a result of prolonged contact with insecticide. There was also a change in the content of any communication, including the prevention aspects of Covid-19. Advocacy at the highest level was organized towards the multi-sector committee of the response to the Covid-19 at the level of the Presidency of the Republic and with the provincial governor. In order to reduce the length of time spent with each household, the NMCP and its partners agreed to reduce the amount of data to be collected by the registration officer.

The information collected before the changes introduced to limit Covid-19 transmission was: (1) Household number on the enumeration sheet; (2) date; (3) name of the health zone; (4) name of the health area; (5) Name of the distribution site; (6) name of the village; (7) surname and (8) first name of the head of household; (9) first name of the father of the head of household; (10) surname, (11) first name and (12) signature of community health worker, (13) first name, (14) initial name and (15) sex of each member of the household up to the 9th member if the household has 10 members or more; (16) Number of sleeping spaces, (17) Number of people in the household.

With changes to limit Covid-19 transmission, which was also the first experience for SANRU with digitization, we have dropped: (13) first names, (14) initial names and (15) sex of each member of the household up to the 9th member if the household has 10 or more members. For the community health worker, (10) the last name and (11) first name was no longer filled in when each household visited (for an average load of 350 households in urban areas and 280 in rural areas), but only once on the 1st day when using the smartphone and (12) the signature is no longer required. This reduced the average time from 10 to 5 min spent in a household for data collection.

#### Macro planning

Macro-planning was organized in two phases: a provincial phase and a national phase. The outputs of the workshops were: an implementation plan with a forecast budget that highlighted a rigorous quantification of needs to complete universal household coverage with LLIN, a detailed timeline, a macro-logistic plan with an input deployment plan that took into account the realities of the province, and a communication plan anticipating bottlenecks encountered during the previous campaign.

#### Training

**Fig. 3:**
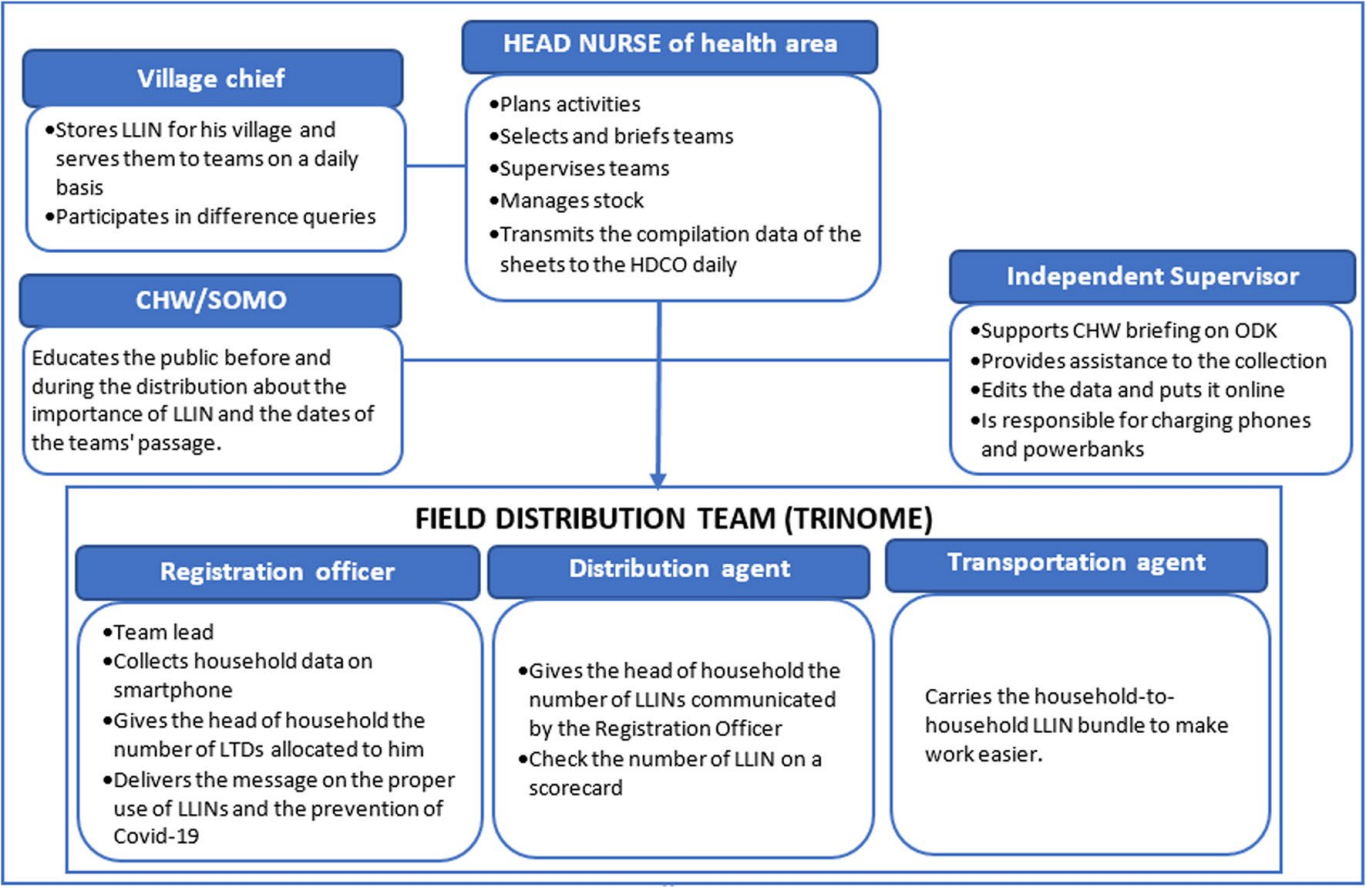
Composition and role of distribution teams as well as that of local supervisors

The training was organized into a series of cascade workshops to strengthen the capacity of all the players involved in the implementation of the LLIN distribution campaign. Between mid-August and mid-October, the cascade training of the actors was organized starting with the Provincial Polyvalent Coaches (PPC), members of the Provincial Executive Team (PET) followed by the Health District Executive Teams (HDET) members of Health District Central Office (HDCO) who then trained the Head Nurses (HN) and the Chairs of the Health Area Development Committees (CHADC). It was these HN and CHADC who were responsible for the recruitment of CHW and briefing them on the eve of the combined registration/distribution under the supervision of the HDETs. They formed the field distribution teams as well as the CHW being responsible for social mobilization (CHW/SOMO). Figure 3 describes the role of field teams and those who provided direct support.

The situation of Covid-19 has imposed special conditions for the organization of training sessions: (i) limiting the number of participants to 20 people, (ii) sharing the revised format of the campaign planning and implementation manual taking into account the change in strategy following Covid-19, (iii) the provision of masks for personal protection as well as bottles of hand gel for hand hygiene, (iv) the arrangement of seats in order to respect social distance, (v) the rooms were deep cleaned before and after each session.

#### Micro planning

From October 28th to 30th 2020, a provincial micro-planning workshop was held to validate the HD microplans. These microplans detailed all the needs required for a successful campaign, procurement plans and roles of the different actors.

Micro-planning resulted in a need for LLIN exceeding the quantity purchased, which included an additional 16% to mitigate stockout risk. Therefore, an adjustment of the distribution key was applied to stay within the envelope of the amount of LLIN available. A choice between two possible distribution keys was made for each health area based on their malaria burden. This has kept a more "generous" distribution key for HD with a high incidence of malaria. All partners appreciated this malaria-led approach that will be maintained for future campaigns.

#### Supply and inventory management

A total of 3,062,850 nets of the Yorkool and Permanet 2.0 brands, all impregnated with deltamethrin, were ordered in January and June 2020 from China and Vietnam respectively. These LLINs were purchased by AMF as part of an agreement between the DRC Ministry of Health, with the distribution costs funded by the GFTAM. Since there was no separate registration, given the Covid-19 context, it was important to ensure that teams had enough nets to distribute to households. This is the reason that an additional quantity of 413,500 nets (included in the total of 3,062,850) was purchased, after analysis performed by SANRU and PNLP looking at household sizes from the previous campaign. This also included enough nets for a 5% buffer at health centres to ensure nets could be moved to where needed. The LLINs provided by AMF were funded with a total participation of 15,714 donations from 6,435 unique donors across 79 countries. In August 2020, the first LLINs were received at the port of Matadi and stored in four central warehouses from 27^th^ August until they were deployed to the HDs from 1^st^ December 2020.

For about 4 months, LLINs were stored in warehouses meeting the following criteria: adequate space for the volume of LLIN, security (presence of a fence, iron door that has not been damaged), waterproofing of the roof, space reserved only for the storage of LLINs and no other products, space being a dry place and sheltered from the elements, good pavement and presence of pallets, good water pipe system around the warehouse, good ventilation system (presence of ventilation holes), accessible to large trucks, having fire extinguishers and thermometers.

The deployment to the HDs was carried out by a single distribution agent selected by SANRU Asbl and lasted about a month except for the Kimvula HD which took two more weeks due to the particularly challenging geography creating logistical difficulties. The LLINs received in the HDs were stored in zonal warehouses selected by the HDET before they were deployed to the villages from 23^rd^ December 2020. At this level several carriers had been involved, selected by the HDET with the support of the PET and the Support and Financial Management Unit (“Cellule d’Appui et de Gestion Finacière” = CAGF) of the MOH.

### Campaign coordination

Coordination is a key factor in the success of the campaign. It was organized at all levels of the health pyramid.

1. At the national level, a National Technical Committee (NTC) met weekly to monitor the implementation of activities, provide feedback to the provinces and prepare key elements to address, if necessary, the decision-making level of the National Minister of Public Health during the meetings of the National Coordinating Committee (NCC), which he chairs weekly to coordinate major public health interventions.
2. At the provincial level, a Provincial Coordinating Committee (CPC) monitored the successful progress of the campaign in the HDs. A total of 10 meetings were held at a rate of two during the preparatory phase, one daily during distribution and two after distribution for an immediate evaluation of the results of the campaign before the final validation workshop, which is expected to take place two weeks after the end of the campaign.
3. At the HD level, the Local Coordination Committees (CLCs) monitored the proximity of the campaign in the HA. The frequency of meetings was the same as that of the CPC with an additional meeting on the catch-up day of missed households.

### Distribution of LLINs to households

The distribution of LLINs to households was launched on 27th December 2020 in the midst of accelerating transmissions of Covid-19 in Kongo Central province, which at that time became the 3rd most affected province. It was carried out door to door for 7 days followed by one to two days of catch up with missed households.

The distribution key (the algorithm for allocating LLINs to households) was based on household size. As mentioned earlier, there were two distribution keys, based on malaria burden of each health zone (Table 1 and Table 2).

**Table 1:** Distribution key for the health zones with the highest malaria burden

Number of people in household	LLINs allocated
1–2	1
3–4	2
5–6	3
7–8	4
> = 9	5

**Table 2:** Distribution key for the health zones with lower malaria burden

Number of people in household	LLINs allocated
1–2	1
3–4	2
5–6	3
> = 7	4

The number of LLINs to be donated was automatically generated according to the size of the households on the ODK form contained in the smartphone. A question confirming the number of LLINs given to the household was introduced in the ODK form including a non-admission constraint of a number different from that generated previously on the form. To ensure that households received the required number, a posteriori check was carried out at the end of the day by comparing the data stored in the smartphone and the tally sheet completed by a 2nd CHW.

Since there was no separate registration, to ensure that teams had enough nets to distribute, HA were provided with LLIN including a reserve. The quantification was based on the population enumerated during the previous campaigns and updated using the annual population growth rate divided by the factor 1.8 (WHO recommendation). An additional quantity was added based on analysis of household size, as mentioned above, which included a 5% buffer to the teams.

An independent check of 5% household audit was conducted to assess and improve the quality of the registration and distribution data. AMF randomly selected the list of villages in each HA and forwarded it to the NMCP before the start of the registration combined with the distribution. During a visit to a selected village that was carried out one or two days after the distribution teams passed, the verification officer passed through 25 randomly selected households using a specific methodology agreed with AMF. In the middle of the village, the officer spun a bottle and went to the first household in the direction indicated by the bottleneck. Then he continued to visit households with a 3-household gap until the 25th household. In each AS, the number of villages to visit was obtained by dividing 5% of the number of micro-planned households by 25. This audit was undertaken by the "National Coordination of the Network of Civil Society Organizations" (CNRSC) which also collected data electronically.

### Digitalization of campaign data management

**Fig. 4:**
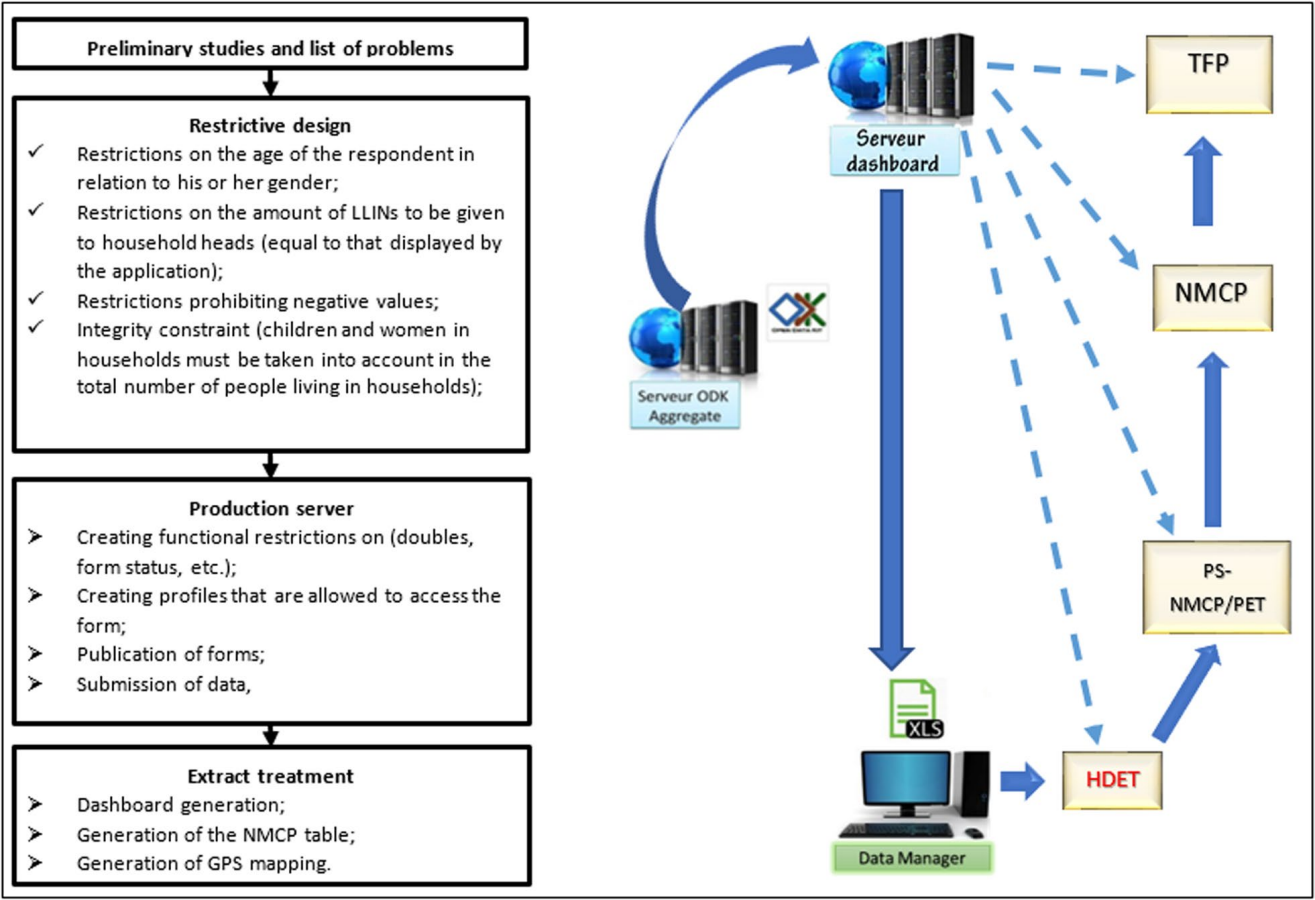
Design and set up the ODK for campaign data management.  Steps in the design and implementation of the application and its deployment on the web; Data flows from field actors to users after validation by NMCP, Access to data for real-time implementation tracking and corrective actions

Prior to the Kongo Central campaign, campaign data management was mainly paper based in the provinces where campaigns were jointly organized by SANRU Asbl and CAGf to support the NMCP agenda with funding from the GFTAM. As part of AMF’s substantial contribution to funding nets for the mass campaign, a requirement was agreed to digitize all distribution data collection and management. SANRU Asbl has developed a software application based on the Open Data Kit Collect (ODK) to meet this challenge. This process (Fig. 4) went through a form design incorporating the Kongo Central province subdivision at three operational levels: HD, HA and villages. Then settings were incorporated into the form to anticipate frequent errors such as the entry of minor respondents taking into account registration gender, different amounts of LLINs than required, negative value entries, incompatibility between the breakdown of household members by category and the total number of household members, etc. The form was coded in XML.

A unique identifier was implemented to avoid double entry of households using the concatenation of character strings linking the Name, First Name and number of people in the household as well as the first name of the head of the household's father.

Configurations at the central server level have consisted of creating restrictions on duplicates, creating user profiles for each form and publishing forms to make them accessible on phones via a QR code.

Data collected in the field by phone was transmitted to a central server. With low network coverage in the province, the system was designed to allow registration agents to collect data offline and upload it to the server later in the day when they could connect to the internet. From the central server, the data was extracted to the "Extract" server where a cleanup (deletion of non-validated data) was done before generating the dashboard, the NMCP output boards and the different maps according to the needs of the users. This allowed stakeholders to use the data on a daily basis for real-time campaign management decision-making.

In order to anticipate the difficulties of this first digitalization pilot, SANRU and the CAGF had organized, in support of the NMCP, a test distribution in 4 HDs in the city-province of Kinshasa, including one completely rural, one semi-rural and two completely urban at the beginning of December 2020. This pilot of about 300,000 households tested the system at scale and recreated the challenges and constraints of an entire province. Lessons learned from the test were studied during an evaluation workshop and the results shared with Kongo Central stakeholders to capitalize on the experience.

To ensure the smooth running of this digitalization pilot in Kongo Central, a highly selective and competitive process was organized to train 566 Independent Supervisors (IS) among 1256 candidates and retain 399 at the rate of one per AS. These IS were responsible for the capacity building aspects of CHW in the use of smartphones as well as for troubleshooting problems in data recording and data management on a day-to-day basis. The session was held concurrently in two cities across the province with up to 29 rooms per location to ensure social distancing with masks and frequent use of hydroalcoholic gel in accordance with the barrier measures enacted by the DRC Government. In order to ensure the consistency of the training, the session was organized via Video Conference Zoom with a facilitator in each room responsible for the management of speeches in connection with the coordination of the training.

**Fig. 5:**
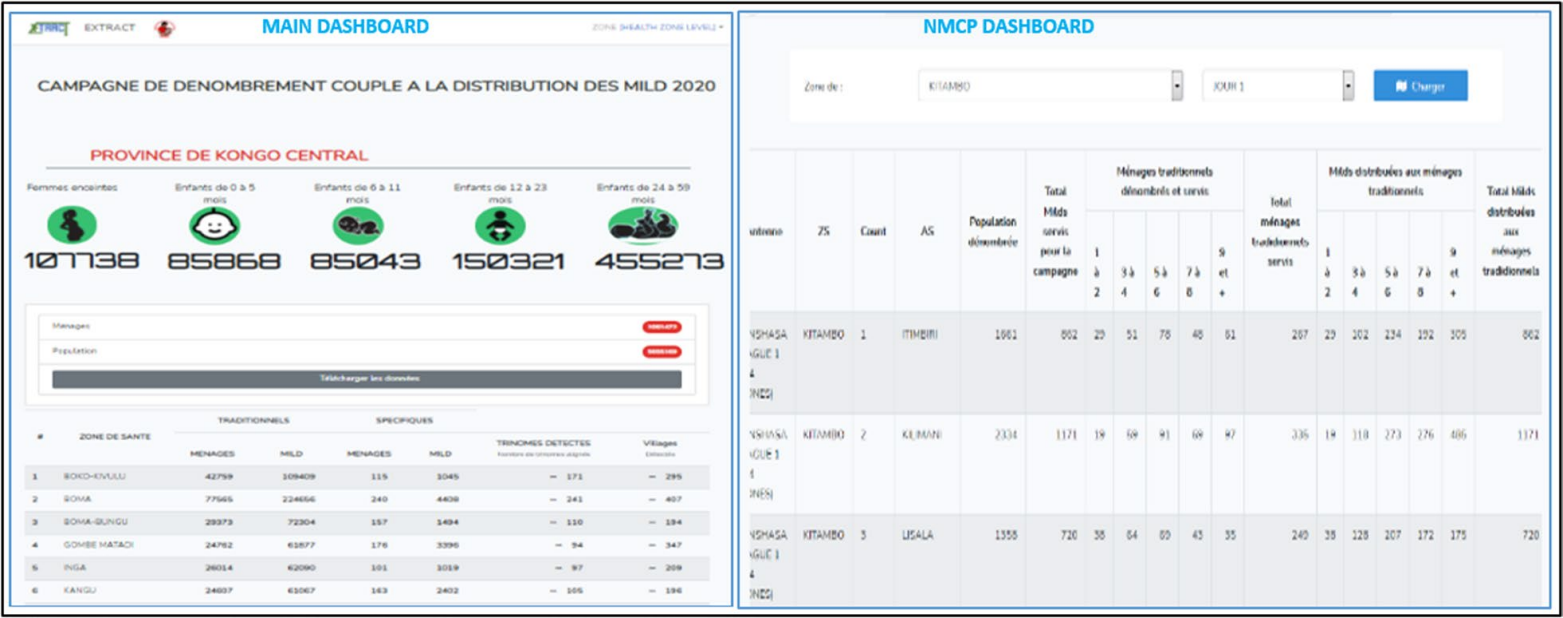
Dashboards available online

A total of 4,467 smartphones were deployed across the province with 2174 power banks to enable household registration in conjunction with the distribution of LLINs. These smartphones are distributed as follows: one per field distribution team, one per 5% household CSO auditors, 4 for HDET members who oversaw locality groups of 3 to 5 HA, one for provincial supervisors deployed at a rate of one per HD, one for provincial inspectors deployed at a rate of one per HD and one for each central supervisor of the NMCP deployed in the province (8). This digitization made the data available on a near real-time basis with two main dashboards (Fig. 5).

A first dashboard is available from the home page after clicking on the "Data" icon and then on the link to a province of your choice. It shows the number of pregnant women, children aged 0 to 5 months, children aged 6 to 11 months, children aged 12 to 23 months and children aged 24 to 59 months. It also shows the total number of population registered and served as well as that of households registered and served. It presents data by HD, the number of households registered and served, the number of LLINs distributed, the number of villages and operational tiers.

A second NMCP dashboard displays the data in accordance with the Excel database used by the NMCP for data validation and sharing with its partners. It can be accessed from the main dashboard display screen, by clicking on "data download" and then on the "Registration combined with distribution" resource, and then on "Statistical Data (PNLP Base)". It is then possible to select the HD and each day of distribution. This allows to have all the indicators collected usually by the NMCP presented by HA: population registered, Total households registered and served as well as breakdown by size and type of household (traditional vs specific). These daily summaries were of significant value to the actors in the field because it allowed them to follow the progress of the campaign in terms of households served and the stock of LLIN as well as some adjustments between health areas.

The following sections present detailed extractable data from the extract server supplemented with data from the daily HN compilation based on scorecards.

## Results

### Demographics

A total of 5,629,211 people were registered in 1,065,537 households, an average of 5.3 persons per household (Table 3). There was a 7.7% increase in the population registered compared to the projected population for micro-planning and a reduction of 6.2% of households registered compared to the number of households projected for micro-planning (Table 3). The distribution of household members by age group recorded a total of 85,872 children under one year of age, 12 to 23 months of 85,047, 24 to 59 months 455,297 and pregnant women 107,739, or 1.53%, 1.51%, 2.67%, 8.09% and 1.91% of the population, respectively. The distribution of households by size of 1–2 people, 3–4 people, 5–6 people, 7–8 people and ≥ 9 persons was 16.9%, 24.2%, 25.8%, 19.7% and 13.4%, respectively.

**Table 3:** Population and households registered compared to projections from micro-plans

Health zone	Health area	Population registration			Household registration			
		Projected population	Population registered	Variation (%)	Projected household	Registered and served	Variation (%)	Households served (%)
Boko Kivulu	20	208,498	228,775	9.73	45,326	42,874	− 5.41	95
Boma	11	380,017	441,874	16.28	82,612	77,805	− 5.82	94
Boma Bungu	12	132,501	139,787	5.50	28,805	29,535	2.54	103
Gombe Matadi	15	116,284	113,904	− 2.05	25,279	24,968	− 1.23	99
No	10	117,777	124,336	5.57	25,604	26,115	2.00	102
Kangu	12	119,918	124,241	3.60	26,069	24 760	− 5.02	95
Kibunzi	10	78 ,701	75,211	− 4.43	17,109	16,647	− 2.70	97
Kimpangu	13	125,437	124,403	− 0.82	27,269	25,269	− 7.33	93
Kimpese	20	272,849	306,889	12.48	59,315	54,738	− 7.72	92
Kimvula	12	89,690	85,271	− 4.93	19,498	16,555	− 15.09	85
Kinkonzi	11	77,898	69,838	− 10.35	16,934	15,912	− 6.04	94
Kisantu	17	317,333	357,756	12.74	68,985	58,755	− 14.83	85
Kitona	6	104,846	109,145	4.10	22 793	22,254	− 2.36	98
Kizu	7	76,924	76,900	− 0.03	16,723	16,447	− 1.65	98
Singing	11	102,376	98,283	− 4.00	22,256	21,904	− 1.58	98
Kwilu Ngongo	21	219,381	242,923	10.73	47,692	43,015	− 9.81	90
Lukula	16	235,685	240,276	1.95	51,236	47,263	− 7.75	92
Luozi	12	100,514	97,213	− 3.28	21,851	20,373	− 6.76	93
Mangembo	10	63,094	55,582	− 11.91	13,716	13,332	− 2.80	97
Time	14	161,445	189,104	17.13	35,097	32,954	− 6.11	94
Matadi	12	511,863	612,412	19.64	111,275	103,194	− 7.26	93
Mbanza Ngungu	12	227,735	262 010	15.05	49,508	43,998	− 1.13	89
Muanda	12	268,674	300,744	11.94	58,407	60,045	2.80	103
Ngidinga	15	147,507	144,410	− 2.10	32,067	28,847	− 10.04	90
Nselo	11	98,034	95,853	− 2.22	21,312	19,738	− 7.38	93
Nsona Mpangu	21	145,778	175,099	20.11	31,691	37,521	18.40	118
Nzanza	10	242,018	261,835	8.19	52,613	45,530	− 13.46	87
Seke Banza	16	189,797	199,024	4.86	41,260	39,854	− 3.41	97
Zna Child	11	114,789	114,552	− 0.21	24,954	21,245	− 14.86	85
Tshela	9	90,595	79,747	− 11.97	19,695	16,903	− 14.17	86
Flash	10	87,767	81,814	− 6.78	19,080	17,285	− 9.41	91
Total Province	399	5,225,725	5,629,211	7.72	1,136,027	1,065,635	− 6.20	94

Out of a total of 3,062,850 LLINs ordered, 3,055,157 were shipped to HDs based on their needs after validation of microplans and 2,886,096 distributed (94% of LLINs ordered). In the end 2,886,623 (94.5%) were distributed to households generating a theoretical remaining stock of 160,171 (Table 4).

**Table 4:** LLIN distributed

Health zone	*Quantity shipped to ZS*	*Amount received by ZS*	*Amount LLIN redeployed*	*Amount distributed*	*Theoretical balance*	*Certified reliquat*	*loss*
Boko Kivulu	109,617	109,617	+ 1168	110,454	331	62	269
Boma	242,219	242,237	−3800	229,065	9372	9316	56
Boma Bungu	72,153	72,153	+ 1750	73,801	102	102	0
Gombe-Matadi	65,284	65,284	+ 1050	65,764	570	388	182
No	61,205	61,205	+ 2300	63,109	396	389	7
Kangu	69,295	69,295	0	63,463	5832	5798	34
Kibunzi	42,690	42,690	0	41,778	912	912	0
Kimpangu	67,644	67,644	0	62,833	4811	4755	56
Kimpese	153,688	153,689	0	147,066	6623	6125	498
Kimvula	46,629	46,629	−527	41,961	4141	4138	3
Kinkonzi	39,393	39,393	0	36,803	2590	2581	9
Kisantu	191,683	191,706	0	169,483	22,223	22,047	176
Kitona	60,902	60,902	0	60,620	282	281	1
Kizu	40,945	40,914	0	39,431	1483	1459	24
Singing	56,500	56,500	−5388	50,437	675	555	120
Kwilu Ngongo	131,448	131,398	0	126,280	5,118	5118	0
Lukula	131,378	131,378	0	120,840	10,538	10,538	0
Luozi	53,488	53,483	0	50,310	3173	3161	12
Mangembo	33,983	33,982	0	32,576	1406	1404	2
Time	97,734	97,737	0	91,905	5832	5797	35
Matadi	344,710	344,710	−8600	316,944	19,166	18,998	168
Mbanza Ngungu	149,756	149,756	−2206	134,449	13,101	12,984	117
Muanda	160,212	160,212	+ 2049	162,080	181	168	13
Ngidinga	79,050	79,050	0	73,188	5862	5674	188
Nselo	53,693	53,678	0	49,070	4608	4602	6
Nsona-Pangu	79,746	79,746	+ 16,900	96,125	521	521	0
Nzanza	158,940	158,943	−10,700	138,384	9859	9496	363
Sekebanza	104,964	104,964	0	99,410	5554	5537	17
Zna-Child	62,917	62,917	0	56,111	6806	6673	133
Tshela	45,853	45,853	0	40,995	4858	4833	25
Flash	47,438	47,438	0	41,361	6077	5759	318
Total Kongo Central	3,055,157	3,055,103	−6004	2,886,096	163,003	160,171	2832

The standard AMF 5% audit of households carried out by the CNRSC showed that 94% of households were actually visited and received nets.

Out of a total of 11,070 villages validated for micro-planning, 10,149 (91.7%) were actually identified and served during distribution (Table 4). This corresponded to a potential saving of $36,840 for the expenses of village chiefs. A total of 3,680 field teams were detected out of 3,947 microplans corresponding to a potential saving of USD 18,690 for expenses of field teams. Calculated on a basis of USD 5/day for 8 days, or USD40 per village chief; Calculated on a basis of USD 5/day for 7 days, i.e. USD35 per Registration Agent and USD 35 per Distribution Agent, the carrier does not influence the calculation because paid in proportion to the bundles transported.

### Mapping the households served

**Fig. 6:**
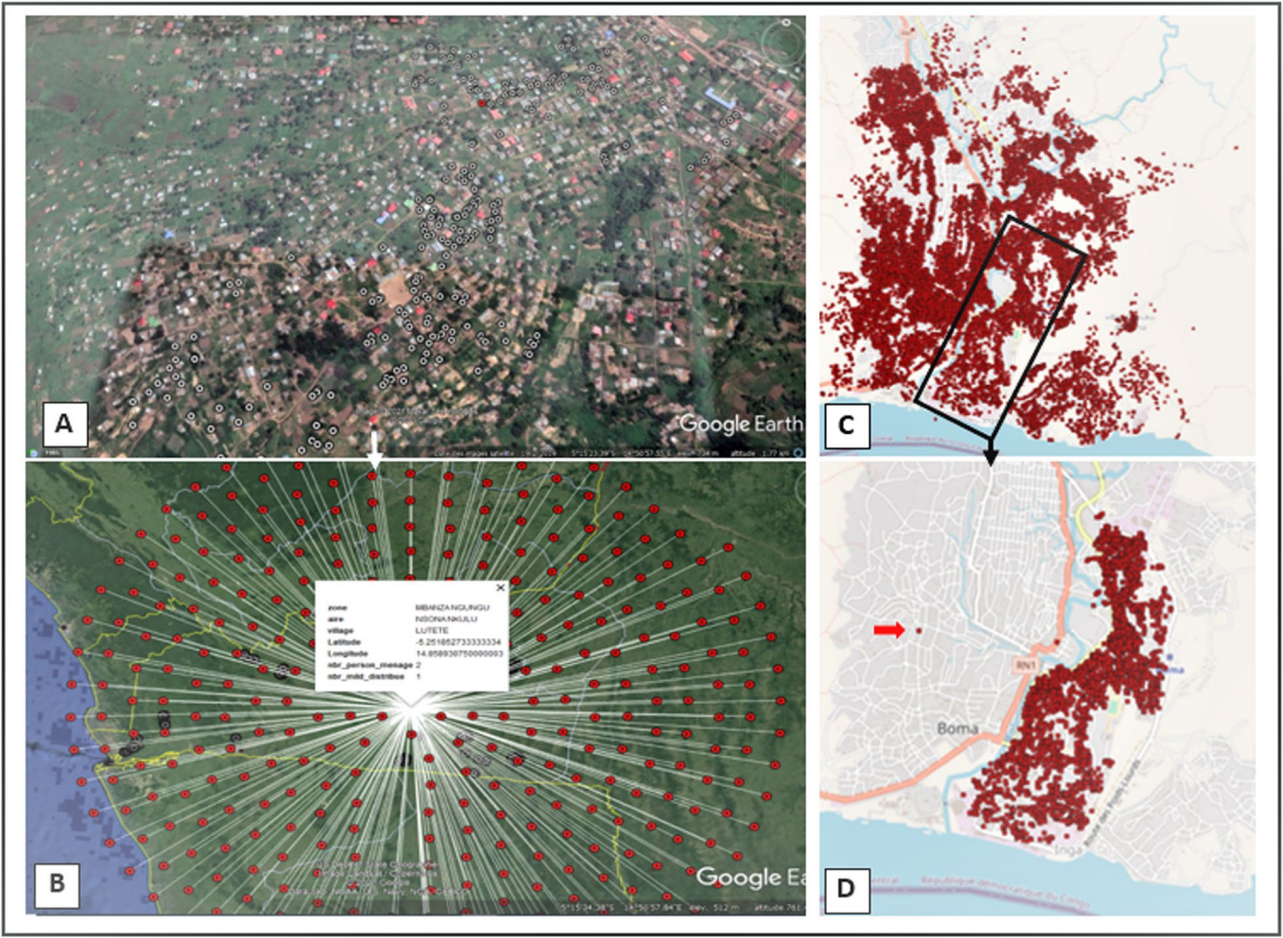
Distribution Maps of households served: (**A**)—Household projections on the provincial map; (**B**)—Pointing at a geographical point that unrolls a household's collected; (**C**)—Household projections on the HD Boma; (**D**)—Household projections on the HA Boma-City with, highlighted with the arrow, a household potentially out of the area

The collection of GPS coordinates generated maps to locate the households served and to analyze the compliance of operations in terms of the complete coverage of local households and the adequacy of the number of LLINs to the number of people in households (Fig. 6). The feature also allowed for simultaneous display of households where the 5% audit took place, as well as supervision at different levels: field teams, HD, province, national.

## Discussion

The LLIN mass distribution campaign in Kongo Central presented immense challenges. Not only was it the first mass campaign to be digitized by the SANRU-CAGF consortium representing the first digital distribution on this scale in this vast country, but it was also the first to be organized after the suspension of campaigns following COVID-19.

The Covid-19 pandemic situation has resulted, among other things, in the distribution of LLINs without prior household registration usually organized to adjust provincial microplans. This is a challenge as the last population census was in 1984 [19]. Registration is essential as there has been intense rural exodus due to insecurity and high unemployment, dilapidated road infrastructure that discourages the predominantly rural population from farming due to lack of evacuation routes so there is a significant and unpredictable fluctuation of the population between the HDs [23]. This largely explains the variation from -13.5% to 17.8% recorded (Table 3) between the projected population from the registration carried out 3 years earlier and that registered during this distribution. The fluctuation observed in Kongo Central (7.7%) is close to that observed in Benin (9%) during a mass distribution organized the same year with, again, a first digitalization experience [24].

In contrast to Benin, where the size of households varied in the same direction as that of the population (up 9% and 13%), the variation in Kongo Central was reversed (up 7.7% and − 6.2%) [24]. This may indicate a propensity to exaggerate the number of people living in the household in order to benefit from more LLINs. The use of digital data collection and management enable possible ways to combat this through data checks and validations, which are being considered for further iterations of the software.

Finally, the quantity of leftover nets of 160,171 LLIN at the end of the campaign (Table 4) suggests that the micro-planning does not always provide reliable data and can overestimate population numbers.

It took a number of measures to meet the challenges posed by this distribution without prior registration. It is in this context that AMF agreed to order a buffer stock of LLIN (5%) beyond that generated by the planning of 1.8 LLIN for two people as recommended by the WHO and the Alliance for Malaria Prevention [25–28]. There was also a strategy consisting of LLIN distribution in all HA by leaving a lagged AS by leaving his stock to be redeployed to the declared rupture sites and then the collection of surpluses in the other HA to finalize the distribution in the lagged HA. The use of the overstock of some HD (4 out of 31 HD) to complete the HD with gap (6 out of 31 HD) was also effective. This was particularly the case for the Sona Mpangu HD which had the largest deficit of 16,900 LLIN (79,900 LLIN microplanned Vs 96.646 LLIN needed) due to the expansion of the border city of LUFU, a place of significant traffic between DRC and Angola. These LLINs had to be redeployed from the surplus stock of Matadi ZS (8,600 LLIN) and Nzanza (8,300 LLIN). The supply time was 48 h thanks to digitization which allowed the Provincial Coordination Committee to decide promptly on the reallocations to be carried out.

Effective coordination between international partners (GFTAM, AMF, RBM Partnership to End Malaria, AMP), NMCP and its partners in the registration (SANRU, CAGF, BDOM-Boma, CNRSC and PNC) and regular communication, notably via a weekly video-conference between the main campaign donors (GFTAM and AMF), the NMCP, SANRU and the CAGF have enabled the rapid resolution of problems such as the acquisition of personal protective equipment (PPE) and the provision by the GFTAM of additional resources as a result of increased training costs and data planning and validation workshops due to Covid-19. Digitalization has also been decisive in the promptitude of corrective actions.

All these factors explain the performance of the results obtained (94% of households registered and served compared to expected households and 94% of LLINs distributed). This performance is identical to that achieved in Benin (94% of the LTDs distributed and 93.4% of the households served) [24].

With a coverage of 94% of household visited and served with LLIN as verified by an independent way namely, the national coordination of the network of civil society organizations using standard AMF 5% audit, this digitalization of LLIN mass distribution has been an important factor in the success of this campaign. In addition to the availability of real-time data for prompt decision-making as discussed above, it has generated efficiencies by detecting discrepancies between important planning elements such as the number of teams and the number of villages (Table 5). The detection of these discrepancies before any payment has enabled the CAGF's finance agency to intensify its audits and make substantial savings.

**Table 5:** Number of villages and planned teams compared to actual and operational numbers during distribution

Health zone	Planed	Number of villages			Number of field teams			Saved Funds (USD)
		actual	Deviations	Saved Funds (USD)	Planed	actual	Deviations	
Boko Kivulu	341	295	46	1840	162	151	11	770
Boma	406	407	− 1	− 40	237	230	10	490
Boma Bungu	254	194	60	2400	103	98	7	350
Gombe Matadi	447	347	100	4000	91	79	14	840
No	140	209	− 69	− 2760	92	87	6	350
Kangu	454	196	258	10,320	94	93	12	70
Kibunzi	227	218	9	360	62	57	15	350
Kimpangu	502	342	160	6400	98	89	16	630
Kimpese	243	483	− 240	− 9600	212	200	11	840
Kimvula	401	353	48	1920	73	63	12	700
Kinkonzi	101	320	− 219	− 8760	61	53	4	560
Kisantu	439	298	141	5640	247	232	8	1050
Kitona	80	82	− 2	− 80	82	77	1	350
Kizu	244	282	− 38	− 1520	60	57	5	210
Singing	205	202	3	120	81	76	6	350
Kwilu Ngongo	491	531	− 40	− 1600	171	151	13	1400
Lukula	685	487	198	7920	184	174	9	700
Luozi	331	255	76	3040	79	68	9	770
Mangembo	237	224	13	520	49	41	10	560
Time	462	384	78	3120	128	117	9	770
Matadi	526	529	− 3	− 120	344	339	7	350
Mbanza Ngungu	526	401	125	5000	177	169	4	560
Muanda	529	329	200	8000	212	202	2	700
Ngidinga	434	553	− 119	− 4760	116	105	13	770
Nselo	362	373	− 11	− 440	79	69	13	700
Nsona Mpangu	292	245	47	1880	116	99	9	1190
Nzanza	347	344	3	120	159	160	22	− 70
Seke Banza	526	468	58	2320	148	131	0	1190
Zna Child	263	240	23	920	90	80	2	700
Tshela	233	223	10	400	71	67	5	280
Flash	342	335	7	280	69	66	9	210
Total Province	11,070	10,149	921	36,840	3947	3680	274	18,690

The mass distribution campaign for LLINs in Kongo Central was originally planned for a fixed site distribution strategy as had been achieved in 2017. However, with the onset of the Covid-19 pandemic, it was not possible to continue with this approach because of the risk of accelerated transmission of Covid-19. A multi-registration analysis of 14 mass distribution campaigns in 5 African registrations showed, after adjusting for other factors, that the delivery strategy (from house to home versus fixed points) and the distribution approach (integrated versus LLIN campaign alone) had no systematic impact on household registration or possession of LLIN [28].

Despite the good results of the mass distribution of LLINs to Kongo Central in the context of Covid-19 transmission, several challenges were encountered in its implementation: (i) the change in approach following the occurrence of COVID-19 from the fixed-site approach to the door-to-door strategy; (ii) the first digitalization of the campaign's data, leading to a new process of capacity building for implementation actors; (iii) the mobilization of PPE for the protection of actors and households, as well as additional resources for the implementation of social distancing measures requiring the reduction of staff in rooms and the increase in the number of rooms, facilitators and extra staff; (iv) agenda conflicts in which the province was engaged in other mass activities such as vaccination and the response to Covid-19 all considered by the MOH to be the highest priority.

SANRU's first experience of digitizing the campaign to support the NMCP has also encountered other challenges such as (i) the province's low electricity coverage mitigate by a loan in powerbanks loans from the EWARS project (Project to digitalize the surveillance of potential epidemic diseases in the preparation phase lead by the MOH Directorate of Disease Control with the financial support of the GFTAM and WHO Country Office); (ii) the province's poor telephone network coverage for uploading data; (iii) the short delay between the testing of the new digital system and the launch of the campaign requiring rapid integration of the lessons learned from the testing; (iv) qualitative variations (differences in the spelling of entities between lists transmitted with village names and those transmitted from the health subdivision e.g.) or the settings transmitted by the PET; (v) the profile of some CHW unsuitable with the ability to use smartphones.

The main lessons learned from this campaign are: (i) digital data management provides quality data for real-time decision-making and efficiencies; (ii) the distribution of LLINs without prior registration can leads to an overestimation of LLIN requirements; (iii) effective stakeholder coordination and political engagement at the top political tier of the province are essential to the success of distribution campaigns.

## Conclusion

Mass distribution campaigns remain the best way to rapidly increase coverage in LLIN, which is a major tool for malaria prevention. This mass LLINs distribution campaign in Covid-19 transmission context in the Kongo Central province, one of the 10 "HBHI" provinces in the DRC, the second most malaria-affected country in the world, is a major response from the DRC to WHO's call to continue with malaria prevention and PEC activities despite the COVID-19 pandemic [12]. An improvement in the digitization of campaign management relating to the quality of training, supervision and logistical flow monitoring will make it possible to take better advantage of this tool in future campaigns.

## Data Availability

The dataset used and/or analysed in this study is available from the corresponding author on reasonable request.
